# Vulnerability of growing cities to solid waste-related environmental hazards: The case of Mthatha, South Africa

**DOI:** 10.4102/jamba.v11i1.632

**Published:** 2019-04-29

**Authors:** Vuyayo Tsheleza, Simbarashe Ndhleve, Hlekani M. Kabiti, Christopher M. Musampa, Motebang D.V. Nakin

**Affiliations:** 1 Department of Biological and Environmental Science, Walter Sisulu University, Mthatha, South Africa; 2 Risk and Vulnerability Science Centre, Walter Sisulu University, Mthatha, South Africa

## Abstract

The rising prevalence of the failure of fast-growing cities’ waste authorities to account for solid waste service function and provide effective solid waste systems poses serious environmental hazards and health risks. Household solid waste mismanagement in Mthatha, a fast-growing city in South Africa with a rapid population increase, is emerging as a major environmental hazard. An effective solid waste audit system could reduce the extent of this problem. This study aimed at categorising and quantifying household solid waste generation and determining the drivers of waste generation and mismanagement that have the potential to increase risk and/or vulnerability to household solid waste-related environmental hazards. Stratified random sampling was used to select 248 sample households and to categorise them according to upgraded high-density informal residential settlements (64), high-density formal residential settlements (62), middle-density residential settlements (61) and low-density residential settlements (61). The results revealed that the waste generation rate increased one moves from informal settlements (1.84 bags of waste per household per week) to low-density, low socio-economic statuses (2.26 bags), middle-density settlements (2.39 bags) and low-density residential settlements (2.84 bags). Food waste was the most commonly generated type of waste for more than 50% of the respondents. Approximately 89% of the most common types of waste reported across all settlements had the potential to be recycled, reused or composted. Only four factors emerged as significant determinants (*p* < 0.05) of the volume of solid waste generated per household per week: household socio-economic status, household size, knowledge of waste management and household participation in waste separation. Results on drivers of household solid waste generation and variations across residential settlements could be utilised when designing growing cities’ waste management plans, with the objective of reducing the volume of solid waste sent to landfill sites, illegal dumping and open burning of waste, thus reducing the associated negative impacts that mismanaged waste poses to the environment. Enforcing waste separation at the household level could promote reuse and recycling, which in turn would reduce waste volumes.

**Keywords:**

household waste generation; socio-economic drivers; residential density; refuse removal; waste types.

## Introduction

Solid waste mismanagement is a growing environmental hazard equally affecting fast-growing cities from both developing and developed countries (Gutberlet 2018). Human activities generate waste and how these wastes are handled, stored, collected and disposed of could pose risks to the environment and to public health (Mohammed & Eyasu 2017). Hoornweg, Bhada-Tata and Kennedy (2013) indicate that globally, the waste generation rate has risen tenfold since the last century and is likely to double by 2025. Solid waste generation in Southern Africa has also escalated as a result of the burgeoning population, rapid urbanisation rates, economic growth and general improvement in living standards (Fuggle & Rabbie 1994; Muzenda 2014). The amount of urban waste produced globally is growing faster than the rate of urbanisation (Hoornweg & Bhada-Tata 2012). Kawai and Tasaki (2016) noted that municipal solid waste generation per capita ranged from 0.09 kg per day to 5.50 kg per capita per day and the median was 0.94 kg per day. In sub-Saharan Africa, South Africa has the third highest municipal waste generation per capita of 2 kg per person per day after the Seychelles and Comoros, with 2.98 kg and 2.23 kg per capita per day, respectively (Kawai & Tasaki 2016). There is a general consensus in the literature that household solid waste generation is directly linked to an area’s socio-economic indicators. Growing cities and cities enjoying positive socio-economic indicators should pay attention to household solid waste generation and management as a way of reducing waste-related environmental hazards. South Africa is already experiencing high waste generation per capita; the reported improvement in its population’s socio-economic indicators and rapid urbanisation are likely going to exacerbate the vulnerability of many cities to waste management problems. Municipalities should therefore prepare their systems for increased solid waste volumes as a way of reducing their vulnerability to waste-related environmental hazards.

It is becoming increasingly expensive for city authorities across the globe to manage solid waste in ways that are environmentally friendly and protective to human health (Gutberlet 2018). A lack of proper waste management systems results in rampant littering as tons of waste end up haphazardly disposed of, posing risks to human health and the environment (Igbinomwanhia 2011; Okot-Okumu 2012). According to Mohammed and Eyasu (2017), waste is dumped on land in an uncontrolled manner, and in most cities openly burning waste, illegal dumping and sending large volumes of waste to landfill sites are common practices. Waste is dumped in the streets and in drains, thus contributing to flooding, the breeding of insect and rodent vectors, the spread of diseases and the uncontrolled release of methane by anaerobic decomposition of waste. Solid waste that has been improperly disposed of is a major source of greenhouse gases that exacerbate global warming (Yadav 2018) and is generated faster than any other environmental pollutants (Hoornweg et al. 2013; Thanh, Matsui and Fujiwara 2010). As waste generation rates across the globe continue to show an increasing trend, waste management authorities, especially in most countries’ fast-growing cities, are bound to fail to account for solid waste service function and to provide effective solid waste systems.

There is a concern that the mismanagement of household solid waste may be a significant risk factor for environmental degradation. The inefficiency of most growing cities’ waste management systems can be partly attributed to insufficient information on waste production, handling and sorting in developing countries, which is difficult to obtain, given the lack of records and often-informal nature of waste management and disposal (Aslani & Taghipour 2018). Quantitative estimation of household waste is needed to estimate the potential and as a basis for a municipal waste management plan. Databases on waste characteristics and generated quantities, including information on drivers, provide credible information for waste managers and planners. Gawaikar and Deshpande (2006), Thanh et al. (2010) and Senzige et al. (2014) highlighted the fact that information about waste characteristics and generated quantities allows for the accurate estimation of resource requirements for collection, transportation, processing and disposal of waste generated in a particular area. Senzige et al. (2014) further stressed that proper information on the composition of waste would also enlighten waste management authorities on potential environmental hazards and existing opportunities for recycling, composting and energy generation, thus reducing the amount of waste that the authorities they have to dispose of in landfill sites. Keeping pace with waste generation challenges requires appropriate schemes (reduce, reuse, recycle – the three Rs) and proper disposal to protect the environment (Ansah 2014). Thanh et al. (2010) emphasised that reliable data on household waste generation is the initial step for the successful implementation of an integrated waste management planning system in any city. To achieve a sustainable solid waste management system, a waste database for a city is required.

Households are at the centre of solid waste generation in any city’s residential areas. Thus, households’ socio-economic factors play a significant role both as risk factors and as key factors in the prediction of vulnerability to solid waste-related environmental hazards and solid waste generation, and waste management behaviour and practices, respectively (Miller & Spoolman 2012; Van Beukering et al. 1999). Households of a similar socio-economic status are likely to have similar waste generation and management characteristics, as reflected by the quantity and composition of waste that a household generates (Pandey, Surjan and Kapshe 2018). Research into the effects of both household demographics and socio-economic factors and their important link with household waste management behaviour is the key to understanding the vulnerability of any city to solid waste mismanagement and environmental hazards. Identifying socio-economic and household demographic characteristics that influence waste generation and disposal patterns is important for waste management planning purposes. This study aimed to categorise and quantify household solid waste generation and determine the drivers of waste generation and mismanagement that have the potential to increase risk and/or vulnerability to the environmental hazards related to household solid waste across communities in Mthatha, as an example of a fast-growing city in Southern Africa. In this study, the drivers of waste generation could equally be referred to as *risk factors*, as mismanaged waste poses an environmental hazard and results in unhealthy living conditions.

In addition to the wide array of international agreements that promote environmental management and sustainable waste management systems, South Africa has its own regulations and policies. The aim is to facilitate sound waste management practices that take into consideration the protection of public health and the reduction of vulnerability of the environment to hazards, by ensuring the sustainable collection, proper treatment and safe disposal of waste. The *South African National Environmental Management Waste Act* (*No. 59 of 2008*) inculcates that sustainable development requires the generation of waste to be avoided, or where it cannot be avoided, that it be reduced, reused, recycled or recovered, and only as a last resort, be treated and safely disposed of. The act also highlights the potential for the utilisation of waste as a resource for creating economic opportunities. Furthermore, the White Paper for Integrated Pollution and Waste Management for South Africa (Notice 227 of 2000) incorporated waste generation, recovery, transportation, treatment and disposal in one plan to facilitate holistic and integrated management systems for pollution prevention and the minimisation of waste at point sources, in order to monitor pollution of the environment.

At a local level, Mthatha city waste management practices are governed by the *Environmental Conservation Act* (*73 of 1998*), which prohibits littering and provides for the authority in control of public spaces to ensure the public areas are free of litter through the provision and discarding of litter bags within a reasonable time. The act is buttressed by the *Municipal Structures Act* (*17 of 1998*), which stipulates that the responsibility for waste management is overseen by the local municipalities. Every municipality in South Africa is required in terms of the *Municipal Systems Act* to prepare its own Integrated Development Plan (IDP). One of the elements within the IDP should be an Integrated Waste Management Plan, which, in terms of the National Waste Management Strategy, must implement the hierarchical management of waste, with emphasis on waste avoidance, minimisation and responsible disposal. The *Municipal Systems Act* also includes recycling as one of the activities to be promoted by municipalities when setting tariffs for waste management services. In Mthatha, waste collection and transportation services are provided by the municipality. The waste is transported to designated dumping sites (DEDEA 2009). However, the municipality does not provide any waste recycling services but rather leaves that role to private companies and individuals. According to Buso, Nakin and Abraham (2014), there exists a disconnect across solid waste management parameters such as distribution, size and type of household waste collection bins, juxtaposed with the waste generation capacity and population density in Mthatha. Although it is the responsibility of the local municipality to provide waste management services, a number of challenges and shortfalls are notable, particularly within rural, formal residential and informal residential settlements (Satterthwaite, Sverdlik & Brown 2018). The segregated provision of waste management services was also noted in a study carried out by Sibanda, Obange and Awuor (2017). Consequently, illegal waste dumping and burning is a prevalent practice in Mthatha (Buso et al. 2014; DEDEA 2009). The provision of services is skewed towards the high-income settlements, while the urban poor are often left with the burden of solid waste management, which exposes them to unhealthy living conditions (Kubanza & Simatele 2016).

## Study area

Mthatha is a city in South Africa that presents a perfect example of a rapidly urbanising city in sub-Saharan Africa. Mthatha is the third largest town in the Eastern Cape province of South Africa and is the only town within a 230-km radius of the Transkei region, serving as an economic and social hub to eight functionally lower-ranked towns in the region and the surrounding rural settlements (ORTDM IDP 2013). From the city centre, Mthatha exhibits layers of both commercial and residential settlement patterns that consist of varying house forms, densities, spaces and differentiated spatial qualities. The general structure of Mthatha is dominated by high-density settlements located on both the eastern and western sides of the city centre, as well as a number of low- and middle-density settlements and spacious peri-urban settlements. The city presents growth trends typical of many cities in sub-Saharan African countries with some sections organised in a regular manner, having their roots in the colonial era, and unregulated areas with informal planning that characterises exponential population growth following independence (Lupala 2002). Urban transformations following independence are usually not adequately supported by infrastructure development and this is common among most fast-growing cities in South Africa, which negatively impacts service delivery. Subsequently, for most cities the quality and distribution of public services and infrastructure like solid waste management facilities, as well as the implementation of domestic solid waste programmes and appropriate solutions to management problems, are complex and vary spatially within the same city. Thus, for every city, effective service delivery requires careful understanding of the city’s development process, spatial distribution of commercial and residential areas (formal and informal settlements) and the area’s population dynamics, as well as the state of and demand for infrastructure.

**FIGURE 1:**
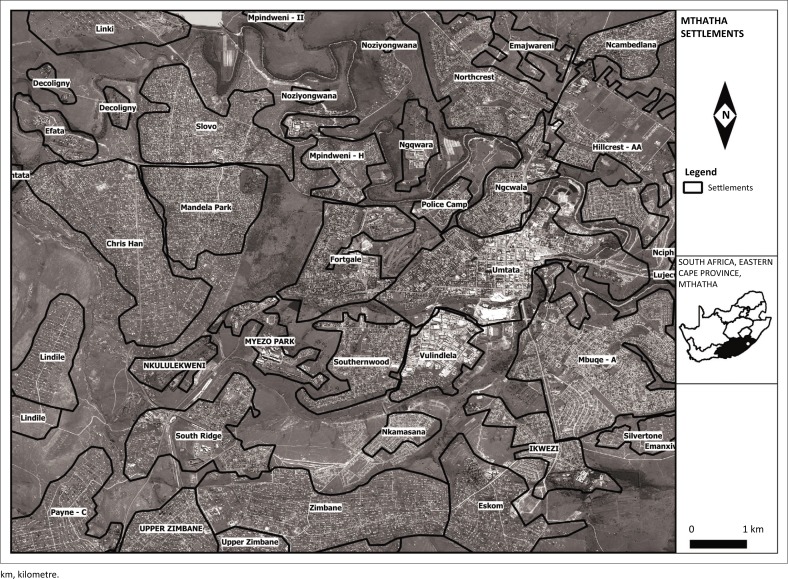
Map showing residential settlements and road network in Mthatha.

Figure 1 shows a map of Mthatha, the residential settlements and the road network.

About 137 589 people live in Mthatha (StatsSA 2012). The town caters for the needs of over 1.5 million people who live within a 230-km radius of the Transkei region (OR Tambo District Municipality ([ORTDM] 2013). Mthatha’s spatial development trends, especially the development of both the informal and formal settlements, and the spatial growth of its peri-urban settlements, reflect most of Southern Africa’s fast-growing cities. The city in its entirety has 3 low-density residential areas, 7 middle-density residential areas, 9 high-density–low-income residential settlements and 11 high-density–low-income informal settlements.

About two-thirds of its citizens live in poverty, with 52% being formally unemployed (Harrison 2008). The educational level of residents is an important determinant of both household waste generation and management. According to the Community Survey (2016), 62.7% of the population in Mthatha has completed Grade 9 or higher. This proportion is higher than the regional percentage of 58% and a little less than the provincial proportion of 64.6%. The town is generally made up of professionals, non-professionals, semi-skilled workers, unskilled workers, business people and the unemployed (Chireshe et al. 2010). The notably higher proportion of the educated population in Mthatha is an important resource for waste management.

Table 1 shows that municipal solid waste collection services in Mthatha favours low- and middle-density residential settlements while neglecting upgraded informal settlements and high-density formal residential settlements. High-density formal and informal residential settlements tend to practise personal removal of waste including open dumping and/or burning of waste (Poswa 2001; Sikrweqe 2013; Stats SA 2012). The principal reasons for neglecting these areas are non-payment of municipal rates and lack of infrastructure, with the latter being the main reason in informal settlements. Recent reports from the Community Survey (2016) show that only 15.5% of households are receiving refuse disposal services from a local authority and 71.0% of the households practise personal removal of waste. Sikrweqe (2013) also noted as a common practice among most municipalities that high-density formal and informal settlements do not receive refuse removal. Municipal authorities’ failure to provide waste collection services to some households is a huge driver of households’ mismanagement of waste. While promoting mismanagement of waste at a household level, the practice results in an increase in illegal dumping sites, which are common in high-density formal and informal settlements. South Africa’s Department of Environmental Affairs (DEA) (2012) reported that every year in South Africa approximately 59 million tons of waste ends up in the environment and only 10% is recycled, posing an environmental hazard. Discriminatory waste collection practices by waste authorities that favour some residential settlements could result in an increase in the number of illegal dumpsites and household burning of waste, thus harming the environment.

**TABLE 1:** Description of waste management practices in the investigated communities.

Residential settlement status	Location name	Area (km^2^)	Number of households	Population density (*p*/km^2^)	Weekly refuse removal (%)	Own refuse removal (%)
Informal settlements	Joe Slovo	4.69	3583	2621.00	0.9	99.1
	Mandela Park	3.32	3347	3448.00	2.2	97.8
High-density	Zimbane	1.24	336	269.00	0.0	100.0
	New Payne	8.13	2659	1260.00	0.7	99.3
Middle-density	Mbuqe Park	3.48	1709	489.02	100.0	0.0
	North Crest	2.11	2138	1011.10	100.0	0.0
Low-density	Myezo Park	1.30	109	83.84	100.0	0.0
	South Ridge	1.93	348	180.42	100.0	0.0

*Source*: Statistics South Africa (Stats SA), 2012, *Census 2011*, viewed 02 April 2015, from https://www.statssa.gov.za/publications/P03014/P030142011.pdf.

## Methodology

A multistage sampling procedure was used in this study. Eight residential areas in Mthatha were categorised according to density, and stratified random sampling was applied. All the residential settlements in Mthatha were categorised into four classes: high-density upgraded informal settlements, high-density formal settlements, middle-density residential settlements and low-density residential settlements. In this study, household density was adopted as a proxy for household socio-economic status. The study focused on the different residential settlements based on density because households from these categories differ significantly in terms of socio-economic status, waste generation, management and municipal provision of services (Senzige et al. 2014). Furthermore, high-density residential settlements were categorised into upgraded informal settlements and formal settlements; this was deemed necessary because these two types of settlements resemble different development processes, which has a strong bearing on municipal delivery of waste management services, infrastructure development and level of organisation. Household waste generation and management and the provision of municipal services to these residential areas differ significantly. Two residential settlements were then randomly selected from the four categories. Approximately 30 households were randomly selected from each location. A house-to-house survey was done systematically using a structured questionnaire to gather data on household socio-economic variables and waste generation. The socio-economic variables of interest in this study were age, gender, household size, employment status, education level and income level.

**FIGURE 2:**
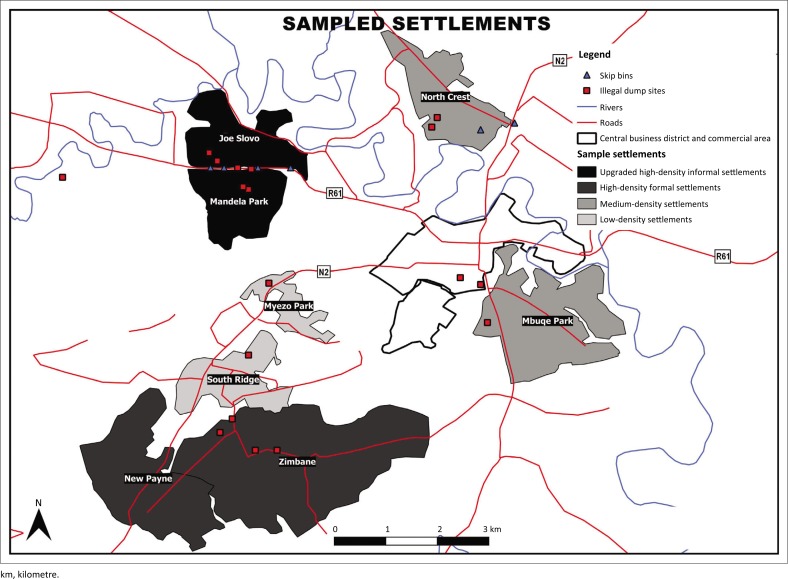
Map showing the investigated communities in Mthatha.

Figure 2 illustrates the investigated residential settlements in Mthatha, grouped into four different formal settlement patterns, the fourth being informal settlements. It also shows the road network, as well as legal and illegal dumping sites spotted by researchers during data collection (the latter represented by red squares).

Low-density residential settlements are inhabited by high socio-economic status households and these have the lowest population density. Subsequent to this group are medium-density residential settlements, whose inhabitants are usually households belonging to the middle socio-economic status category. Both low-density and medium-density residential settlements receive weekly refuse removal from the municipality. The third category is high-density residential settlements. In this study, the high-density residential settlements were further divided into two categories: formal high-density residential settlements and upgraded informal high-density residential settlements. These two harbour the highest population density and their inhabitants have the lowest socio-economic status, relative to low- and medium-density residential settlements. However, unlike high-density formal settlements, informal settlements are unregulated and generally excluded from public-sector resources. Even where the city government chooses to provide door-to-door waste collection services to these settlements, it is impractical because of infrastructural challenges, and where possible it is reportedly very irregular and unreliable. It is interesting to note that most upgraded informal settlements lack the infrastructural capacity for waste management. Thus, cities require a different waste management strategy that addresses the demands of upgraded informal settlements differently. Therefore, analyses of this nature that seek to note differences in waste management practices between high-density formal residential settlements and high-density upgraded informal settlements will likely produce results that are suitable for scaling up and replication. In many cities it is common for 30% – 60% of the urban population to live in informal settlements (Campos & Zapata 2014). Informal settlements represent a universal phenomenon that many countries suffer from, and it is one of the major phenomena accompanying the accelerated urbanisation process worldwide (Khalifa & Khalid 2014).

Data was entered in Microsoft Excel and subsequently transferred to SPSS (version 17) (Chicago, IL, United States) for statistical analysis. Proportion, mean and standard deviation (SD) were calculated for all households’ socio-demographic information. Data on household solid waste generation (household waste characterisation and quantification) was split into four groups (upgraded high-density informal settlements, low-income, middle-income and high-income households). Following this, a multiple logistic regression analysis was performed to determine the drivers of household solid waste generation. Lastly, multiple comparison analyses were employed to detect any statistically significant difference in terms of number of bags of solid waste generated per household per week between the four socio-economic statuses.

## Results and discussions

### Household demographics

Table 2 shows the summarised socio-economic demographic characteristics of the sampled households categorised into informal settlements, low-income, middle-income and high-income households. Household socio-economic demographic characteristics are important in waste management as they influence the type and quantity of waste generation and its overall management in any setting (Etengeneng 2012; Parfitt, Flowerdew & Doktor 1994; Van Beukering et al. 1999).

**TABLE 2:** Household demographics for selected communities – Socio-economic demographics.

Household demographic variables	Upgraded high-density informal settlements (%) (*N* = 64)	Formal high-density residential settlements (%) (*N* = 62)	Middle-density residential settlements (%) (*N* = 61)	Low-density residential settlements (%) (*N* = 61)
**Gender of head of households**				
Male	36.00	40.00	38.00	31.00
Female	64.00	60.00	62.00	69.00
**Age**				
15–30 years	27.00	35.00	21.00	13.00
31–45 years	48.00	40.00	33.00	25.00
46 and above	25.00	24.00	46.00	62.00
**Household size**				
Mean	4.42	6.25	5.13	4.49
SD	2.31	2.56	1.78	1.10
**Educational level**				
No formal education	13.00	8.00	2.00	2.00
	22.00	19.00	3.00	0.00
Secondary	39.00	39.00	23.00	15.00
Tertiary	27.00	34.00	72.00	84.00
**Employment status**				
Employed	47.00	37.00	48.00	54.00
Unemployed	27.00	29.00	15.00	5.00
Self-employed	16.00	24.00	15.00	33.00
Pensioner	8.00	2.00	18.00	8.00
Student	3.00	8.00	5.00	0.00
**Income level**				
Below R2500.00	47.00	50.00	25.00	2.00
R2500.00 – R10 000.00	41.00	19.00	23.00	2.00
Above R10 000.00	12.00	31.00	52.00	96.00

Table 2 shows that female-headed households were more common than male-headed households (all above 60%) in all four categories, with low-density residential settlement households showing the maximum percentage of 69%. These results are comparable to Sikrweqe (2013) and show consistency with the national norm (Stats SA 2012). Female-headed households are dominant across most communities in South Africa. Using gender of head of household as a proxy indicator for household involvement in waste management, Mattos, MacKinnon and Boorse (2012) found that there is a relatively higher participation in waste management among female-headed households than male-headed households. The domination of female-headed households in Mthatha may undoubtedly and positively contribute to waste management at large. The four communities are therefore expected to generate less waste and manage it in a sustainable manner.

Mean household size for the four residential areas ranged from four to six members per household. Households from formal high-density residential settlements and middle-density residential settlements had the highest household sizes as compared to their counterparts in upgraded informal and low-density settlements. Household heads aged 46 years and above dominated middle-density and low-density residential settlements. The dominant age group in high-density and informal settlements was 31–45 years of age (see Table 2). As expected, respondent households from low-density and middle-density residential settlements were more educated; 87% and 72% respondents had attained tertiary certificates, respectively. Respondents with secondary education dominated in informal and formal high-density residential settlements. According to Etengeneng (2012), households with higher levels of education tend to have a more positive attitude towards waste management. Conversely, households with a lower level of education tend to be ignorant or unaware of the impact of generating huge volumes of waste and its effects on the environment (Parfitt et al. 1994). Informal settlements (27%) and middle-density settlements (48%) had the highest percentage of unemployed respondents. The level of unemployment definitely implies that there is a possibility of labour that can benefit solid waste recycling activities. Waste recycling can help to reduce waste volumes directed to landfill sites and increase the lifespan of a landfill (Adogu et al. 2015). Thus, the observed variations across the four residential settlements in terms of socio-economic indicators are expected to have a significant impact on household solid waste management practices, thus impacting the city’s vulnerability to environmental hazards.

### Waste characterisation and quantification

Household solid waste includes many different waste types and a number of household products that can pose a risk to the environment and human health. Thus, the necessity of an assessment of household solid waste composition concerning the presence of waste types that could pose risk to the environment cannot be overemphasised. Furthermore, the characterisation and quantification of waste can contribute to proper decision-making for the solid waste strategy (three Rs) of a city as knowledge on these key ingredients determines the required investment for the city (Chung 2015; Gawaikar & Deshpande 2006).

#### Generated waste types

The household waste composition data presented in Table 3 shows the proportion of different types of waste found among the investigated households. Households’ solid waste per week was dominated by food waste. Food waste (55%) was the most common type of waste reported across all the sampled households. Respondents from informal settlements (14.1%) reported papers and plastics as the highest contributors to their waste. Nineteen per cent of the households in informal settlements reported that disposable nappies were the highest contributor to solid waste.

**TABLE 3:** The most common types of waste according to socio-economic status – Characteristics of generated waste.

Waste types	Upgraded informal residential settlements (%)	High-density residential settlements (%)	Middle-density settlements (%)	Low-density residential settlements (%)	All households (%)
Food waste	46.9	38.7	65.6	70.5	55.2
Plastic	14.1	12.9	9.8	3.3	10.1
Papers	14.1	12.9	11.5	4.9	10.9
Tins and metals	6.3	25.8	4.9	1.6	9.7
Glass	0.0	3.2	1.6	8.2	3.2
Disposable nappies	18.8	6.5	6.6	11.5	10.9

A bigger proportion of waste types collected in all four settlement categories has a potential to be recycled. This presents a possible recycling opportunity and strong case for promoting markets for waste recycling. However, there was no evidence of separation at source; household waste with the potential of being recycled was mixed with other waste types in one bag, taken by a collection truck and sent to a sanitary landfill site. Waste separation at source has the potential to improve household solid waste recycling and thus increase municipal solid waste management efficiency (Mian et al. 2017). To improve recycling and reduce waste transported to landfill sites, Mian et al. (2017) recommend that recycling and separate collection of waste be included as part of municipal responsibilities. Source separation of households’ solid waste improves the proportion of waste recycling.

With regard to food waste, the results are similar to the study conducted by Ojeda-Benitez, De Vega and Ramí (2003), Asare, Andrews and Asare (2015) and Qu et al. (2009), who found that food waste is the highest contributor to solid waste as compared to all other domestic wastes. Food waste falls under organic waste. The studied communities could benefit through composting, thus reducing waste volumes transported to landfill sites. According to Ojeda-Benitez et al. (2003), a high volume of organic waste presents an opportunity for recycling through organic waste composting. High volumes of food waste and other organic waste can negatively impact the environment. Untreated and unmanaged food waste creates odour and hygiene concerns and causes adverse environmental impacts (Khoo, Lim & Tan 2010). Thus, the high composition of organic waste in the generated household waste presents a significant waste hazard if the waste is not properly managed or sent to landfill sites. Biodegradation of household solid waste produces acidic and alkaline organic pollutants and other pathogens with the ultimate production of leachate with heavy metal, which causes serious surface and ground water contamination (Khoo et al. 2010). Different waste types present varying environmental impacts if not managed well or when sent to landfill sites; the same waste types present an important economic opportunity if suitable treatment or conversion options are undertaken. Thus, a significant reduction of environmental impacts can be realised by implementing recycling and reuse processes. Glass was recorded as the lowest contributor to solid waste, with only 3%. This is comparable to a study by Qu et al. (2009), in which glass accounted for the least generated waste type. In addition to these findings, illegal open dumping of solid waste is the most common practice for the disposal of waste in upgraded informal settlements (see Figure 2).

#### Generated waste quantities

Understanding the waste volume generated at the household level is important for efficient waste management practices, especially with regard to collection. Household solid waste generation is a core indicator of environmental pressure, and this is usually measured in weight or volume (Kawai & Tasaki 2016). Following the volume-based waste accounting system, the recommended number of municipal-sized bin bags per household per week is three bags. Table 4 presents the reported average number of bags generated per household per week in line with the volume-based waste accounting system. The reported maximum number of bags generated per household per week was six for all socio-economic status in Mthatha.

**TABLE 4:** Number of municipal-sized bags generated per household per week in Mthatha, South Africa (*N* = 248).

Socio-economic status	*N*	Maximum	Mean
Upgraded high-density informal settlements	64	6	1.84 ± 1.21
High-density settlements	62	6	2.26 ± 1.08
Middle-density residential settlements	61	6	2.39 ± 1.21
Low-density residential settlements	61	6	2.84 ± 1.32

*N*, number.

The reported average number of bags per household per week showed a slight increase upon moving from high-density informal settlements (1.84 bags) to low-density residential settlements (2.84 bags). This means that in this study, households from low-density residential settlements generated higher volumes of waste than their counterparts. Similarly, Moftah et al. (2016) reported that in Tripoli City, Libya, the majority of households in low-density residential settlements generated high volumes of solid waste. Low-density residential settlements are usually inhabited by households of a higher socio-economic status. Thus, a conclusion can be made that waste volumes increase from low to high socio-economic status households. Therefore, municipal solid waste management strategies and systems should take note of households’ socio-economic status and important residential settlement boundaries.

#### Comparison of different residential settlements

The mean average number of bags of solid waste generated per household per week showed a positive correlation with a slight increase as we move up the four socio-economic statuses shown in Table 5. Thus, socio-economic status has an influence on waste generation. There was a statistically significant difference between the average number of bags of waste generated per household per week across the four socio-economic statuses as determined by one-way analysis of variance (ANOVA) (*F* = 7.349, *p* = 0.000). Furthermore, a Tukey post hoc test revealed that the number of waste bags generated per household per week was significantly lower for households from high-density formal residential settlements (*p* = 0.026) and high-density upgraded informal settlements (*p* = 0.000) when compared to households from low-density residential settlements. There was no statistically significant difference between volumes of solid waste generated per household per week between residents of (1) middle-density and high-density residential settlements and (2) low-density and middle-density residential settlements. The identified differences in volumes of waste per household per week between high-density settlements and low-density residential settlement have important implications for effective waste management.

**TABLE 5:** Summary of statistics comparing the quantity of solid waste generated per household per residential settlement in Mthatha, South Africa (*N* = 248).

Average quantity of waste generated per household per week (bags/week)	Difference†	Std. error	*p*
Middle-density residential settlements–low-density residential settlements	0.1676	0.2160	0.865
Middle-density residential settlements–upgraded high-density informal settlements	0.5497	0.2144	0.053**
Low-density residential settlements–middle-density residential settlements	0.4426	0.2169	0.176
High-density residential settlements–upgraded high-density informal settlements	0.3821	0.2135	0.281
Low-density residential settlements–high-density formal settlements	0.6103	0.2160	0.026*
Low-density residential settlements–upgraded high-density informal settlements	0.9923	0.2144	0.000*

* , Statistical significance at the 10% confidence level.

** , Statistical significance at the 5% confidence level.

† , Difference between quantity of solid waste generated per household per residential settlement.

Similar findings regarding an increase in waste generation from high-density residential settlement to low-density residential settlement households were reported by Ojeda-Benitez et al. (2003) and Asase (2011). Affluence results in high levels of consumption and the waste of resources (Miller & Spoolman 2012). At a household level, the increased standards of living result in increased consumption and this increases the amount of waste generated (Van Beukering et al. 1999). Miezah et al. (2015) and Moftah et al. (2016) found that middle-class communities generated less waste than low-class communities.

#### Drivers of waste generation

Relative to households in upgraded informal settlements, Table 6 shows that households in low-density residential settlements had a significantly higher chance of generating more bags of waste. The household size positively affected the number of bags generated per household. However, households with a higher number of males (*B* = 0.211, CI = 0.082 ± 0.340, *p* ≤ 0.001) had a higher chance of generating more bags of waste than those with a higher number of females (*B* = 0.138, CI = 0.046 ± 0.230, *p* ≤ 0.004). Knowledge of waste management and waste separation practises decreased the number of bags generated (*B* = -0.434, CI = -0.756 ± 0.112, *p* ≤ 0.008 and *B* = -0.313, CI = -0.633 ± 0.007, *p* ≤ 0.055, respectively).

**TABLE 6:** Multiple regression analysis of drivers of household waste generation in Mthatha, South Africa (*N* = 248).

Household demographic variables	*B*	95% CI	*p*
**Constant**	2.386	1.076 ± 3.696	0.000
**Gender of head of household**^†^	−1.112	−0.418 ± 0.193	0.469
**Socio-economic status**			
Informal settlements	-	-	-
High-density settlements	−0.090	−0.532 ± 0.351	0.687
Middle-density settlements	0.234	−0.228 ± 0.697	0.319
Low-density settlements	0.661	0.134 ± 1.188	0.014**
**Age**	−0.074	−0.278 ± 0.130	0.477
**Household size**			
Male	0.211	0.082 ± 0.340	0.001*
Female	0.138	0.046 ± 0.230	0.004*
**Educational level**	−0.015	−0.215 ± 0.185	0.881
**Employment status**‡	0.188	−0.040 ± 0.215	0.170
**Income level**	0.091	−0.147 ± 0.329	0.453
**Knowledge on waste management§**	−0.434	−0.756 ± 0.112	0.008*
**Practising waste separation¶**	−0.313	−0.633 ± 0.007	0.045**

CI, confidence interval; *B, Beta* coefficient.

* , Statistical significance at the 1% confidence level.

** , Statistical significance at the 5% confidence level.

† , male – 1; female – 2.

‡ , unemployed – 1; employed – 2.

§ , no – 0; yes – 1.

¶ , no – 0; yes – 1.

Wealthier individuals are likely to throw away more plastic, metallic, glass and hazardous waste than poor individuals (Chandra & Devi 2009). Affluence, associated with people in high-income areas, causes an increase in the consumption of goods and services and this results in increased proportions of disposable materials, especially packaging materials (Medina 2010; Van Beukering et al. 1999).

A significant positive relationship between household size and waste generation is not peculiar. High household size implies a higher level of waste generation (Agbesola 2013; Ansah 2014). Further inferential analyses show that households with more males generate more waste than female-dominated households. Males and females have different attitudes and perceptions towards public health and household cleanliness. Women are usually responsible for the salvaging of waste (Lutui 2001). Information on demographic distribution, especially household size and household gender distribution, is crucial when planning the waste management activities of any city.

A knowledge of waste management decreases waste generation at the household level. An awareness refers to the ability of the household’s inhabitants to correctly manage the generated waste at the household level and to convert it to a valuable resource (Ojeda-Benitez et al. 2003). These households tend to have a more positive attitude and practice towards waste management because of their increased knowledge of waste issues (Etengeneng 2012). An awareness of waste management is key to alleviating waste management challenges in many cities. Thus, environmental education that promotes waste separation, reuse, recycling and waste reduction should be prioritised.

Households practising waste separation understand the importance of minimising waste. Source separation enhances the recycling of valuable materials, protecting recyclables from contamination and promoting usability. This reduces the number of waste bags generated at the household level (Fiehn & Ball 2005). Source separation relieves pressure on scarce raw materials and reduces environmental problems (Fiehn & Ball 2005; Kamara 2009).

## Conclusion

An inadequate account of waste types, volumes and drivers of household solid waste generation are some of the challenges faced by growing cities in their pursuit to improve waste management systems and reduce the environmental impacts posed by the mismanagement of waste. This study outlined household solid waste generation rate per week, waste types and household socio-economic drivers of waste generation per week. Different residential settlements, categorised by density, generate varying volumes of solid waste. Household demographic factors such as income, household size, education and gender composition play a key role in the determination of the amount and types of solid waste generated per household per week. The results of drivers of household solid waste generation and variations across residential settlements could be utilised when designing growing cities’ waste management plans with the objective of enhancing efficiency and reducing solid waste going to landfill sites. City waste management policies should take cognisance of the residential settlement-related waste variations in addressing associated challenges. Enforcing waste separation at the household level could promote reuse and recycling, which in turn would reduce waste sent to landfill sites and reduce the negative environmental impacts posed by solid waste.
